# Measuring the multidimensional reputation of a medicines regulatory agency: development and validation of a public-oriented scale

**DOI:** 10.3389/fmed.2025.1570817

**Published:** 2025-04-30

**Authors:** Kyung-Bok Son

**Affiliations:** College of Pharmacy, Hanyang University, Ansan, Republic of Korea

**Keywords:** medicines regulatory agency, reputation, development scale, validation, South Korea

## Abstract

**Objective:**

The reputation of public agencies, encompassing the dimensions of performance, morality, procedure, and technical competence, is fundamental to understanding their behavior. However, standardized, individual-level measures of reputation suitable for surveys targeting the general public are lacking. This study aims to develop and validate a survey instrument for the general public to measure the multi-dimensional reputation of public agencies, with a focus on South Korea’s medicines regulatory agency.

**Methods:**

Survey items were developed based on previous literature, refined through expert consultation, and validated through a population survey. The validation study involved 1,000 participants from the public, selected using a quota sampling method stratified by age, sex, and region, according to the South Korean census. Validity was assessed through exploratory factor analysis and hypothesis testing, while reliability was evaluated using internal consistency.

**Results:**

Exploratory factor analysis identified a three-dimensional structure of reputation, encompassing performance, procedure, and technical competence, while morality was not distinctly identified as a separate dimension. Construct validity, including convergent and discriminant validity, was confirmed. The internal consistency of the three dimensions was acceptable, with Cronbach’s alpha coefficients ranging from 0.87 to 0.91. The overall reputation of the medicines regulatory agency was measured at 72 out of 100. The specific dimension scores were as follows: 74 for technical competence, 71 for performance, and 70 for procedure.

**Conclusion:**

The agency should recognize the multidimensional nature of reputation and foster an environment that enables the public to observe and evaluate these dimensions. Reputation management strategies should emphasize not only technical expertise but also performance and procedural aspects to ensure a well-rounded reputation.

## Introduction

Reputation refers to the image of an organization as perceived by diverse audiences. It is defined as “symbolic beliefs about an organization—its capacities, intentions, history, mission—and these images are embedded in a network of multiple audiences (1).” Reputation carries different meanings in the private and public sectors (2, 3). In the private sector, reputation focuses on performance aspects such as competitive advantage or profitability (4). In contrast, reputation in the public sector extends beyond performance to include procedural and moral dimensions (5).

A distinct characteristic of bureaucratic reputation is its multidimensionality (5, 6). This reflects the ambiguity of public organizational goals and the challenges these organizations face (7, 8). Public organizations must address a wide range of interests, which are critical in shaping reputation. While classical literature on bureaucracy highlights expertise, derived from information asymmetry, as a source of bureaucratic power (9, 10), bureaucratic reputation is not confined to expertise alone. Carpenter categorizes reputation as extending beyond expertise to include performative, technical, legal-procedural, and moral dimensions (1). Performative reputation is shaped by perceptions of an organization’s decision-making and effectiveness in achieving objectives, while technical reputation concerns its scientific, methodological, and analytical capacities. Legal-procedural reputation is based on adherence to accepted rules and norms, and moral reputation stems from value-driven and ethical behaviors that generate emotive judgments from the public.

Organizational reputation has emerged as a significant topic in bureaucratic studies over the past decade (11). Building a strong reputation is a critical element of regulatory power and plays a key role in understanding the functions of public administration. Carpenter emphasized that reputation “shapes the power of government organizations, and more broadly, the powers of the state (1). Bureaucratic reputation also serves as an established framework for studying public sector organizations and is utilized as an essential factor in understanding regulation and the behavior of regulators. Numerous empirical studies have demonstrated that reputation is a crucial determinant of bureaucratic behavior (12–14).

Thus, reputation is a key concept in the study of public sector organizations; however, its conceptual ambiguity and multidimensionality present significant challenges for accurate measurement (6, 15). In the private sector, where reputation often focuses on performance metrics such as profitability, a variety of measurement tools are available (16). However, in the public sector, tools for measuring reputation remain limited. Recently, Lee and Van Ryzin proposed an indicator to measure the reputation of public agencies from the perspective of citizens (17). Nonetheless, this indicator has a limitation in that it does not fully capture the multidimensionality of reputation. Building on this, Overman et al. developed a tool to measure the multidimensional reputation of public agencies by incorporating the perspectives of various stakeholders (6).

Several issues related to medicines regulation have emerged as critical concerns (18, 19). Ensuring expedited approvals while safeguarding patient safety are key objectives of medicines regulatory agencies (20–22). To achieve these conflicting goals, agencies have implemented expedited approval programs (23–25) and, more recently, have actively incorporated real-world data/evidence into regulatory decision-making processes (26–29). The successful implementation of these initiatives is closely linked to the reputation of the organization. This study aims to develop and validate a survey instrument for measuring the multi-dimensional reputation of public agencies from the perspective of the general public, with a focus on South Korea’s medicines regulatory agency.

## Methods

We developed the survey items based on a literature review, refined them using expert feedback, and validated the instrument through a population survey.

### Literature review

Following Carpenter’s four-dimensional conceptualization of reputation (1), we generated an initial pool of survey items through a comprehensive review of the literature on bureaucratic reputation. Studies focusing on aspects of corporate reputation, such as prices, profits, and investments, were excluded. This process identified two key papers (6, 17), leading to a total of 40 survey items.

### Interviews with experts

The preliminary questionnaire was assessed for face validity through semi-structured, face-to-face interviews. We evaluated the relevance, comprehensibility, acceptability, and feasibility of the questionnaire using cognitive interview techniques, including think-aloud and probing methods (30, 31). Three experts participated in the assessment: two academics specializing in regulatory science and public health—one of whom had experience working at the Ministry of Food and Drug Safety (MFDS) for one and a half years—and an employee of a pharmaceutical company with over a decade of experience in regulatory affairs. The interviews, conducted in January 2024, each lasted approximately 1 h. The insights gained from these interviews were instrumental in finalizing the questionnaire. Three laypersons evaluated the appropriateness and clarity of the survey items that were finalized based on expert interview results.

### Population survey

#### Participants and sample size

The study population consisted of South Korean public aged 19 years or older. The sample size (*n* = 1,000) was determined using a 95% confidence interval, a 3.1% margin of error, and a standard deviation of 0.5%. A quota sampling method was employed, stratifying the sample by sex, age, and region according to South Korean census data, and the required number of completed surveys for each quota was obtained. The survey was conducted by the agency Realmeter between April 20 and May 6, 2024. Invitations were randomly sent to the pre-registered individuals at the agency’s mobile phones until the target number of completed surveys was reached. Participants were informed of the study’s objectives in the invitations, and those who consented were directed to an encrypted website to complete the survey. Upon completion, participants received a voucher valued at US $4. The study protocol was reviewed and approved by the Institutional Review Board of Hanyang University (HYUIRB-202403-028).

#### Questionnaires and scoring

The final questionnaire comprised four dimensions, each with 10 survey items, addressing performance, morality, procedure, and technical competence. Responses were recorded on a 5-point Likert scale, ranging from 1 (strongly disagree) to 5 (strongly agree), with an additional option of 6 (do not know). Since this study aims to measure the reputation of regulatory agencies among the general public, option 6 was included to ensure the suitability of the survey items for lay respondents. Scores were calculated as the mean of the summed scores for each dimension, excluding responses marked as 6. In addition to the 40 items related to reputation, six other variables were included to test construct validity: budget, autonomy, performance, favorability, political orientation, and socio-economic status. These variables were also measured using a 5-point Likert scale, with an additional option of 6 (do not know).

### Exploratory factor analysis

#### Item analysis

We analyzed the distribution, kurtosis, and skewness of responses for each item. Items that were deemed challenging for the public to answer were excluded. Items with “do not know” (answer 6) responses exceeding 7% or “neutral” (answer 3) responses exceeding 35% were considered unacceptable and were subsequently removed.

#### Exploratory factor analysis

Following item exclusion, exploratory factor analysis (EFA) was conducted to confirm the underlying factor structure of bureaucratic reputation. Sampling adequacy was assessed using Bartlett’s Test of Sphericity and the Kaiser-Meyer-Olkin (KMO) test to determine whether the data were suitable for factor analysis (32). Parallel analysis was employed to determine the number of factors to retain, keeping only those with eigenvalues greater than 1.0 (33). EFA was performed using varimax rotation and the maximum likelihood method. A stepwise approach was applied to enhance factor loadings and minimize cross-loadings, with factor loadings above 0.5 deemed satisfactory.

#### Construct validity

For construct validity, correlations between reputation and six additional variables (budget, autonomy, performance, favorability, political orientation, and socio-economic status) were assessed. We hypothesized strong relationships between reputation and variables such as budget, autonomy, performance, and favorability (convergent validity), and weak correlations with political orientation and socio-economic status (discriminant validity). Spearman’s rank correlation coefficient were calculated, and the strength of correlations was assessed using Cohen’s criteria: strong (*r* > 0.5), moderate (0.3 < *r* ≤ 0.5), and weak (*r* ≤ 0.3) (34).

#### Reliability

Cronbach’s alpha was used to assess the internal consistency of each factor, with values greater than 0.7 considered acceptable (34).

## Results

### Refined items

Following the interviews, most items were deemed suitable for the South Korean context. However, some items within dimension A (performance) were revised. Experts emphasized the importance of consistency, predictability, and responsiveness in MFDS decisions, recommending the inclusion of questions addressing these aspects. Based on their input, questions A2, A3, A4, and A7 were revised. Three laypersons indicated that the finalized survey items were clear and appropriate.

### Item analysis

Supplementary Table 1 presents the characteristics of the study participants. Of the 9,874 invitations sent, 1,000 individuals completed the survey, resulting in a response rate of 10.12%. Table 1 presents the distribution of responses to the 40 items in the final version of the questionnaire. After item analysis, 5 items from dimension A: performance (A1, A3, A4, A7, A10), 6 items from dimension B: morality (B2, B5, B6, B7, B9, B10), 6 items from dimension C: procedure (C2, C3, C7, C8, C9, C10), and 2 items from dimension D: technical competence (D4, D5) were removed. The remaining 21 items were deemed acceptable for exploratory factor analysis.

**Table 1:** Answer distribution for the 40 survey items in the questionnaire.

Dimensions		Survey items	Strongly disagree	Disagree	Neutral	Agree	Strongly agree	Do not know	Skewness	Kurtosis
A: Performance	A1	The Agency has sufficient capacity resources, personnel, capital to deliver on its mandate.	14	74	325	367	118	102	−0.30	3.00
	**A2**	**The Agency responds well to the risks of food and pharmaceuticals.**	19	75	305	385	166	50	−0.41	2.97
	A3	The agency’s decisions are consistency.	22	104	321	359	122	72	−0.33	2.83
	A4	I am satisfied with the service provided by the Agency.	26	82	356	359	125	52	−0.35	3.07
	A5	Replacing the Agency with any other organization would deteriorate the current level of service quality.	25	79	248	389	195	64	−0.58	3.02
	**A6**	**The Agency has a lot of added value.**	20	62	295	383	181	59	−0.46	3.08
	A7	The Agency’s decisions are predictable.	19	106	350	326	113	86	−0.21	2.79
	**A8**	**The Agency makes good decisions.**	22	88	322	379	129	60	−0.38	2.99
	**A9**	**The Agency is a competent regulator.**	27	95	328	335	149	66	−0.33	2.81
	A10	The Agency communicates well with stakeholders.	19	114	370	311	95	91	−0.15	2.82
B: Moral	**B1**	**The Agency’s mission is ethically defensible (their mission is the right mission).**	26	76	340	354	149	55	−0.37	3.01
	B2	The way in which the Agency works is ethically defensible.	28	94	348	342	117	71	−0.33	2.97
	B3	Outputs (e.g., decisions, rules, opinions, products) of the Agency are ethically defensible.	32	88	346	349	122	63	−0.38	3.03
	B4	The Agency has a positive influence on society.	21	56	286	396	197	44	−0.52	3.18
	B5	The Agency shows compassion toward people or organizations that are disadvantaged by its actions.	34	148	344	288	97	89	−0.17	2.63
	B6	The Agency has integrity.	42	130	377	267	95	89	−0.18	2.79
	B7	The Agency works transparently.	38	124	372	275	101	90	−0.19	2.80
	B8	Confidentiality is an important value for the Agency.	22	63	242	381	239	53	−0.62	3.09
	B9	The Agency pays attention to public opinion.	36	110	379	309	101	65	−0.27	2.93
	B10	The Agency is independent from political considerations.	54	171	299	288	111	77	−0.20	2.40
C: Procedure	**C1**	**Decision-making in the Agency follows due process.**	22	74	328	386	140	50	−0.41	3.10
	C2	The Agency works fairly and does not make arbitrary decisions.	32	97	335	345	121	70	−0.37	2.94
	C3	The Agency has a good procedure for complaints.	16	54	311	411	134	74	−0.43	3.32
	**C4**	**The Agency uses all of the relevant evidence.**	24	67	303	407	151	48	−0.51	3.24
	**C5**	**The Agency follows correct procedures.**	25	79	325	378	130	63	−0.42	3.10
	**C6**	**The Agency’s experts are objective.**	31	92	320	354	140	63	−0.40	2.92
	C7	The Agency strikes a good balance between transparency and confidentiality.	29	110	332	329	122	78	−0.30	2.79
	C8	Most opinions by the Agency do not get challenged.	22	159	348	286	101	84	−0.07	2.52
	C9	The Agency is independent from industry.	26	131	323	307	126	87	−0.21	2.58
	C10	The Agency is good at responding to requests for information in an orderly and timely manner.	34	90	355	332	126	63	−0.35	2.98
D: Technical competence	**D1**	**The Agency’s employees are highly skilled in their profession.**	15	44	277	403	205	56	−0.49	3.19
	D2	The Agency’s employees understand the problems and issues in the field.	28	95	336	370	109	62	−0.40	3.04
	**D3**	**The Agency cooperates well with experts in the field.**	17	55	274	431	181	42	−0.54	3.30
	D4	The Agency is a learning organization.	19	91	330	361	119	80	−0.32	2.92
	D5	The Agency has good leadership.	27	83	377	321	117	75	−0.27	3.04
	D6	Expertise in the Agency is well managed, even if an employee leaves.	22	72	283	398	175	50	−0.51	3.08
	**D7**	**The Agency has the capacity to maintain qualified staff.**	18	64	264	434	175	45	−0.56	3.26
	D8	The Agency sets new scientific standards.	22	72	340	373	145	48	−0.37	3.06
	**D9**	**The Agency is at the forefront of scientific innovations.**	15	48	287	404	182	64	−0.46	3.20
	**D10**	**Opinions by the Agency influence what we do in our own organization.**	11	37	230	415	257	50	−0.59	3.25

The items underlined were selected following the item analysis. The items highlighted in bold and underlined were finalized after item analysis and factor analysis.

### Exploratory factor analysis

EFA was conducted on a sample of 752 participants, excluding 248 participants who selected “do not know” (answer 6) for the remaining 21 items. The initial test for sampling adequacy indicated that the sample was suitable for factor analysis (Bartlett’s Test of Sphericity: *p* < 0.0001; Kaiser-Meyer-Olkin (KMO) test = 0.98). Parallel analysis revealed a three-factor structure.

Table 2 presents the results of the EFA, including factor loadings greater than 0.5. Through a stepwise approach, seven items (A5, B3, B4, B8, D2, D6, and D8) were removed. The analysis identified three dimensions: A: performance, D: technical competence, and C: procedure. Out of four items initially expected to load onto the B: morality dimension, three were excluded, and one was reassigned to the dimension C: procedure during the stepwise approach to finalizing the model. The three dimensions partially reflect Carpenter’s four-dimensional conceptualization of reputation. The dimensions accounted for 22, 21, and 21% of the total variance, respectively. Cronbach’s alpha values for technical procedure, performance, and competence were 0.88, 0.87, and 0.91, respectively.

**Table 2:** Exploratory factor analysis with three dimensions structure.

	A: Performance	D: Technical competence	C: Procedure
A8	0.74		
A9	0.69		
A2	0.66		
A6	0.62		
D9		0.64	
D1		0.60	
D10		0.59	
D3		0.57	
D7		0.56	
C5			0.68
C1			0.63
C4			0.61
C6			0.58
B1			0.52
Reliability: α	0.88	0.87	0.91
SS loadings	3.07	2.98	2.87
Proportion variance	0.22	0.21	0.21
Cumulative variance	0.22	0.43	0.64

Supplementary Table 2 presents the distribution of responses for additional survey items included in the questionnaire to assess construct validity. Table 3 presents the correlation coefficients between the three dimensions and integrated reputation, as well as other variables, including budget, autonomy, performance, favorability, political orientation, and socio-economic status. Performance and favorability showed strong associations with the reputation dimensions, with correlation coefficients ranging from 0.67 to 0.78, demonstrating convergent validity. In contrast, political orientation and socio-economic status exhibited weak associations with the reputation dimensions, with correlation coefficients ranging from −0.11 to 0.11, indicating discriminant validity. Autonomy and budget showed moderate associations with the reputation dimensions, with correlation coefficients ranging from 0.45 to 0.52.

**Table 3:** Correlation analysis.

	Autonomy	Budget	Favorability	Performance	Political orientation	Socio-economic status
Dimension A: Performance	0.47***	0.45***	0.67***	0.68***	0.11**	−0.11**
Dimension C: Procedure	0.51***	0.48***	0.67***	0.70***	0.09*	−0.07*
Dimension D: Technical competence	0.48***	0.47***	0.71***	0.75***	0.10**	−0.10*
All dimensions: Reputation	0.52***	0.50***	0.74***	0.78***	0.11**	−0.10**

*p < 0.05, **p < 0.01, and ***p < 0.001.

**Figure 1:**
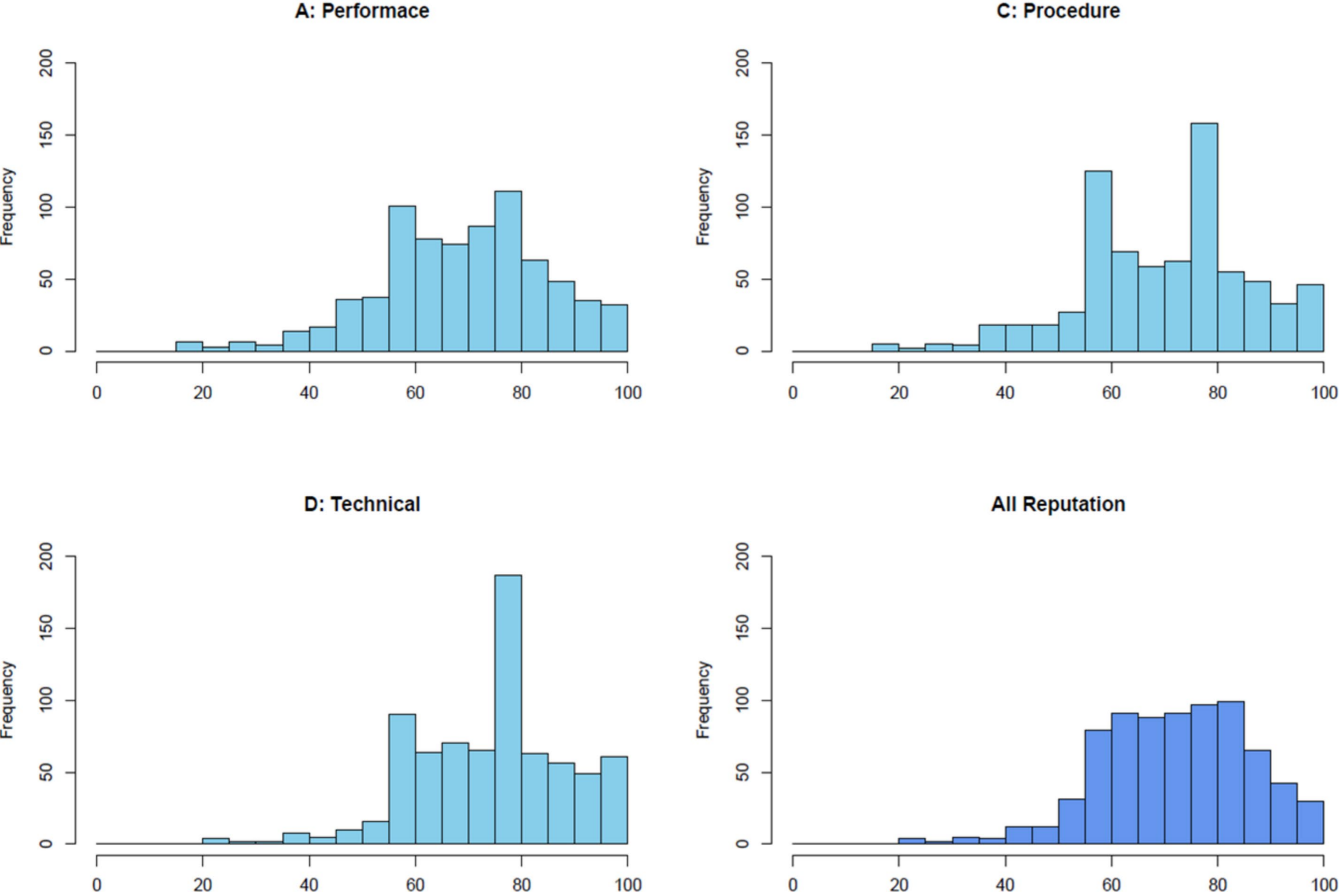
The figure shows the distribution of reputation scores across three dimensions—performance, procedure, and technical competence—as well as the overall reputation. Each histogram represents the frequency distribution of scores converted to a 0–100 scale.

Figure 1 illustrates the distribution of reputation scores across three distinct dimensions—Performance, Procedure, and Technical Competence—as well as the integrated reputation score. Each histogram displays the frequency of scores within a range of 0 to 100. The integrated reputation scores reveal a bell-shaped distribution centered around 72, reflecting an overall positive yet diverse evaluation of reputation. The scores for each dimension were as follows: technical competence scored 74, performance 71, and procedure 70.

## Discussion

This study, building on Carpenter’s framework (1), developed a tool for the general public to measure the “reputation” of a medicines regulatory agency, categorized into four dimensions: performance, morality, procedure, and technical competence. The survey items were refined through interview with experts, item analysis, and factor analysis, and various tests confirmed that the tool demonstrated construct validity and reliability.

### Interesting findings

This study revealed several interesting findings. First, from the public’s perspective, the technical dimension is relatively easier to evaluate, whereas other dimensions, such as performance, morality, and procedure, are more difficult to assess. Second, the factor analysis identified three key dimensions of reputation: performance, technical competence, and procedure. Meanwhile, survey items related to morality were excluded during the analysis process, with one item being integrated into the procedure dimension. Third, using the proposed tool, the reputation of a medicines regulatory agency was measured at 72 out of 100. The specific dimension scores were as follows: 74 for technical competence, 71 for performance, and 70 for procedure.

### Developing a reputation measurement tool for the public

The specialized nature of regulatory agency tasks raises questions about whether the general public is adequately equipped to evaluate such institutions (35). Unlike industry professionals or regulated entities, the public often lacks direct interactions with regulatory agencies, making assessments more challenging (13). The study findings highlight this difficulty: 19 out of 40 proposed survey items were excluded during item analysis, particularly those outside the technical dimension. This outcome suggests that the public, especially those without insider knowledge, struggles to evaluate procedural and moral aspects of medicines regulation, likely due to the abstract or highly technical nature of these concepts.

Audiences play a critical role in measuring reputation. Organizations operate within broad and diverse audience networks, including regulatees, industry professionals, and the general public, each of whom perceives regulatory performance differently (15). The evaluator’s proximity to the organization significantly influences both the feasibility and outcomes of reputation assessments. Those with direct regulatory experience may assess agencies based on compliance and efficiency, whereas the general public may rely on indirect perceptions shaped by media coverage or publicized policy decisions.

Public evaluations remain crucial as medicines regulation ultimately serves society, making the public broader stakeholders (36). The legitimacy of bureaucratic organizations hinges on public support, which is essential for policy credibility. Restricting evaluators to direct stakeholders alone may lead to risks such as regulatory capture or public alienation due to excessive regulatory specialization (37). Despite certain challenges, public evaluations of regulatory agencies remain both justified and meaningful.

### Dimensions of reputation

The public identified technical competence, performance, and procedure as distinct dimensions but did not recognize morality as a separate one. Technical competence was perceived as a clear and distinct dimension, aligning with the understanding that regulatory agencies are tasked with approving drugs based on scientific evidence (38, 39). Moreover, the public differentiated between the technical processes involved in drug approval and the outcomes of those processes, treating them as separate dimensions. However, morality was not distinctly identified as an independent dimension; rather, some of its aspects were integrated into the procedural dimension. This phenomenon may be attributed to the overlap among dimensions and the inherent distance between the evaluator and the evaluated.

The first reason is the overlap between dimensions of reputation (40). The dimensions of reputation are interrelated, and some may overlap with each other (15). During the operationalization of conceptual components, overlaps between certain dimensions may be further strengthened. In fact, a study by Overman et al., which measured the reputation of regulatory agencies, found that the reputation of the European Chemical Agency, as perceived by various stakeholders, consisted of the dimensions of performance, procedure, and morality, excluding technical competence (6).

The second factor is the distance between the evaluator and the evaluated (13). Minimal distance is necessary to assess potentially overlapping dimensions of reputation (5). Stakeholders closely connected to an organization can more clearly identify different dimensions. However, this study evaluated regulatory agencies from the public’s perspective. The relatively large distance between evaluators and the evaluated may have led to the omission of certain dimensions. Notably, this distance does not exert a one-way influence. Public perceptions of regulatory agencies differ from those of stakeholders, often emphasizing different aspects.

### Practical implications for medicines regulatory agencies

The findings of this study provide practical implications for medicines regulatory agencies. First, regulatory agencies should clearly define the dimensions of reputation and establish conditions that enable the public to observe and evaluate these dimensions. Enhancing transparency in decision-making processes and presenting regulatory outcomes in a format that is easily accessible to the public are crucial. In this regard, regulatory agencies in countries with well-established regulatory systems publish Public Assessment Reports (PARs) to promote transparency and facilitate public understanding (41–43). These strategies can clarify the role of regulatory agencies and foster greater public recognition of the value and importance of public organizations.

Second, regulatory agencies should reassess their strategies for improving reputation. While the agency currently focuses on enhancing technical competence and expertise, this study suggest that the public values not only technical competence but also performance and procedural aspects as key components of reputation. The agency should take proactive measures to strengthen its reputation across all these dimensions. Due to the nature of regulatory agencies, moving beyond conventional bureaucratic practices is essential. Instead, adopting flexible policy operations and establishing collaborative frameworks with stakeholders are critical to achieving a more comprehensive and balanced reputation.

One way to implement such a flexible and collaborative approach is by leveraging international frameworks such as the WHO Global Benchmarking Tool (GBT) (44). GBT provides a structured methodology for assessing regulatory maturity, ensuring that agencies meet global standards in governance, technical competence, and procedural efficiency (45). By systematically evaluating key regulatory functions, GBT helps agencies identify and address gaps that could undermine public trust (45). Adopting GBT can serve as a concrete strategy for improving regulatory reputation.

A key advantage of GBT is its role in facilitating regulatory reliance, enabling agencies to build credibility by aligning with internationally recognized standards. Agencies that achieve Maturity Level 3 (ML3) or higher in GBT assessments are acknowledged as competent regulatory bodies, reducing redundant assessments and expediting decision-making processes (46). This shift not only enhances technical capacity but also reinforces procedural transparency (47)—both of which, as this study highlights, are crucial for public reputation. By integrating GBT into strategic planning, regulatory agencies can go beyond technical improvements and establish a more holistic and credible reputation.

### Study limitations

This study has several limitations. First, as it employed a survey-based approach, this study cannot rule out the possibility of selection bias, meaning that survey respondents may have been skewed toward groups with specific characteristics. Second, as the survey was conducted using a self-administered format, some items may have been difficult for respondents to understand, potentially leading to variations in how the survey questions were interpreted. Third, to validate the survey instrument, the study focused on a single agency. Consequently, the findings may have limited generalizability to other types of regulatory agencies. Fourth, perceptions of regulatory agencies are shaped by the experiences and expectations of their audiences. As such, even for agencies of similar types, reputation assessments may not be universally applicable due to variations in national contexts. Fifth, public evaluations of regulatory agencies may be shaped by specific events. Although no major regulatory issues related to the agency occurred before or after this study was conducted, the potential impact of such events should be considered when interpreting the findings.

## Conclusion

This study developed and validated a survey instrument to measure the multidimensional reputation of public agencies, with a focus on South Korea’s medicines regulatory agency. The findings revealed that the agency’s reputation was composed of three dimensions: technical competence, performance, and procedure. The theoretical dimension of morality was only partially represented, being integrated into the procedure dimension. The agency should recognize the multidimensional nature of reputation and foster an environment that enables the public to observe and evaluate these dimensions. Reputation management strategies should emphasize not only technical expertise but also performance and procedural aspects to ensure a well-rounded reputation.

## Data Availability

The raw data supporting the conclusions of this article will be made available by the authors upon reasonable request.
