# Supplementary material for: Measuring the multidimensional reputation of a medicines regulatory agency: development and validation of a public-oriented scale
Source: Front Med (Lausanne). 2025 Apr 30;12:1570817. doi: 10.3389/fmed.2025.1570817 (PMC12075541; doi:10.3389/fmed.2025.1570817)
Supplement: Supplementary file 1 [file Table_1.DOCX]

Supplementary Table 1. Baseline characteristics of participants

| Characteristic | Study population (n=1000) |
| --- | --- |
| Age |  |
| 20s | 157 (15.7%) |
| 30s | 149 (14.9%) |
| 40s | 186 (18.6%) |
| 50s | 198 (19.8%) |
| Sex |  |
| Female | 503 (50.3%) |
| Male | 497 (49.7%) |
| Region |  |
| Metropolitan | 504 (50.4%) |
| Urban | 196 (19.6%) |
| Rural | 300 (30.0%) |

Supplementary Table 2. Answer distribution for the other survey items in the questionnaire for testing construct validity

|  |  | 1  Strongly disagree  Strongly left  The lowest | 2  Disagree  Left  Lower | 3  Neutral  Neutral  Middle | 4  Agree  Right  Higher | 5  Strongly agree  Strongly right  The highest | 6  Don’t know |
| --- | --- | --- | --- | --- | --- | --- | --- |
| Autonomy | The Agency should have more budget. | 25  (2.5%) | 57  (57.0%) | 260  (26.0%) | 402  (40.2%) | 203  (20.3%) | 53  (5.3%) |
| Budget | The Agency should have more autonomy. | 31  (3.1%) | 75  (7.5%) | 335  (33.5%) | 337  (33.7%) | 161  (16.1%) | 61  (6.1%) |
| Favorability | I am favorable to the Agency. | 29  (2.9%) | 84  (8.4%) | 347  (34.7%) | 358  (35.8%) | 146  (14.6%) | 36  (3.6%) |
| Performance | The Agency is performing well. | 22  (2.2%) | 84  (8.4%) | 325  (32.5%) | 393  (39.3%) | 127  (12.7%) | 49  (4.9%) |
| Political orientation | My political orientation is as follows. | 38  (3.8%) | 135  (13.5%) | 625  (62.5%) | 146  (14.6%) | 56  (5.6%) | - |
| Socio-economic status | My socio-economic status is as follows. | 92  (9.2%) | 310  (31.0%) | 480  (48.0%) | 107  (10.7%) | 11  (1.1%) | - |
